# Comparative Evaluation of Iron Deficiency among Obese and Non-obese Children

**Published:** 2014-12-10

**Authors:** MR Sharif, M Madani, F Tabatabaie

**Affiliations:** 1 Trauma Research Center, Kashan University of Medical Sciences, Kashan, Iran.; 2 Student Research Committee, Kashan University of Medical Sciences, Kashan, Iran; 3 Isfahan Endocrine & Metabolism Research Center, Isfahan University of Medical Sciences, Isfahan, Iran.

**Keywords:** Obesity, Micronutrient, Iron deficiency, Children, Ferritin

## Abstract

**Background:**

Obese children are at the risk of micronutrient deficiency especially iron deficiency. Given the importance of iron deficiency in this age group and considering the existing discrepancies, present study was performed with the aim of evaluating iron deficiency in obese children.

**Material and Method:**

This study was conducted, in the form of a case-control study, on 100 children aged between 5 to 15 during June and November 2013 in Kashan. Cases consisted of 50 obese children and controls were 50 children who were not obese.Blood sample was drawn to check for serum iron, total iron binding capacity (TIBC) by biochemistry method and plasma ferritin by ELISA method. The obtained data were entered into SPSS software version 16 and statistically analyzed. P < 0.05 was identified as statistically significance.

**Results:**

The mean values of serum iron were 52.38 and 64.50 microg/dL for the case and control groups (P<0.02).

TIBC levels in obese and non-obese Children were 434.80 and 382.28 microg/dL (P<0.008). The ferritin level in the study group was 70.56 versus 68.06 ng/ml in the control group (P=0.79). In the obese children group, 8.3% (n=2) of children with iron deficiency had ferritin levels less than 15 while in control group 100% (n=14) of iron deficient children had ferritin levels below 15 (P<0.001).

**Conclusion:**

In our study, mean serum iron levels were lower among obese children in comparison with control group. However, ferritin concentrations were similar in both groups. It is supposed that due to inflammatory state caused by obesity, serum ferritin levels are naturally higher in obese people. It is suggested that higher levels of ferritin considered as normal reference values in obese people.

## Introduction

Obesity is a global health issue and in recent years its prevalence has increased dramatically (1,2) to the extent that 16-31% of children and adolescents are said to be obese (2,3). Obese children and adolescents are not only prone to develop complications such as high blood pressure, dyslipidemia and type 2 diabetes mellitus (3), but also tend to consume a diet high in calories and low in nutritional value and are thus at increased risk of micronutrient deficiency especially iron deficiency (4,5). Iron deficiency is considered to be the most common micronutrient deficiency (1) and the major cause of anemia worldwide (6). The negative consequences of iron deficiency anaemia on cognitive and physical development of children, and work productivity of adults are of major concern (7). Iron deficiency in children and adolescents is associated with learning and behavioral disorders (1). It has been estimated that the prevalence of iron deficiency anemia among Iranian children under 5 years old is 18-38% (8). It is obvious that, iron deficiency per se is even more frequent than iron deficiency anemia (9). In a study by Karen et al. on a rather large sample of children between 2 to 16 years of age, overweight children suffering from the highest prevalence of iron deficiency and overall, the prevalence of iron deficiency increased as BMI increased (10). Another study in Mashhad which was performed with the aim to figuring out the association between obesity and low levels of micronutrients such as iron showed that serum iron was low in 56.1% of obese children while 10.4% of children with normal weight had low levels of serum iron (11). However a study in Yazd which was conducted to assess the relation of body mass index with hemoglobin and iron parameters showed that there was no difference in iron deficiency indices between normal weight, overweight, and obese persons (12). In another study in France evaluated the association between micronutrients such as iron and body mass index, no relationship was found between obesity and iron concentrations (13). Given the increasing prevalence of obesity among children and adolescents as well as the importance of iron deficiency in this age group, and considering the existing discrepancies, present study was performed with the aim of evaluating iron deficiency in obese children compared with non-obese subjects.

## Methods and Materials

This study was conducted, in the form of a case-control study, on 100 children aged between 5 to 15 during June and November 2013. The participant divided in two groups of 50 obese children who had admitted to the pediatric endocrinology clinic and control group who were 50 non-obese children referred to the pediatric clinic in Kashan and had no underlying diseases. After examination of children, their BMIs were calculated. Body Mass Index (BMI) is a number calculated from a child's weight and height. It is a reliable indicator of body fatness for most children and teenagers. Children who had BMIs less than 95^th^ percentiles were considered as non-obese and those with BMIs equal to or greater than 95^th^ percentile were classified as obese based on the 2000 CDC Growth Charts (14). Those who had iron deficiency anemia, taking supplements containing iron, had an underlying condition affecting hemoglobin levels, or any infections were excluded from the study. After obtaining written informed consent and reassuring parents that all children’s results would be kept confidential, 5cc blood sample was drawn to check for serum iron, total iron binding capacity (TIBC) by biochemistry method and plasma ferritin by ELISA method. Serum iron levels between 50 to 120 micrograms per deciliter and TIBC values between 240 to 450 micrograms per deciliter were considered as normal ranges. Serum iron levels less than 50microg/dL and TIBC higher than 450 microg/dL were defined as iron deficiency (15). To adhere to the ethical issues, parents whose child had iron deficiency were then informed.

**Statistical analysis**

After data collection, they were entered into SPSS software version 16 and statistically analyzed. The normality of the variables was analyzed using Kolmogorov-Smirnov test. Chi-square test and t-test were also used. P < 0.05 was identified as statistically significance.

## Results

This study comprised 100 children, 50 in the obese children group and the other 50 in control group. In the case group 28 were males and 22 were females. In the other group 23 were males and 27 were females. We found no statistically significant gender differences between the groups (p=0.42). The mean age of the studied children was 9.44±2.59 with the minimum age of 5 and the maximum age of 15. The mean age of children in the study group was 9.50±2.80 years versus 9.38±2.39 years in the control group. Considering the mean age, there was no significant difference between the study groups (p=0.81). In case group, 24 children (48%) were iron deficient and 26 (52%) were normal. While, in control group, 14 children (28%) were deficient. In this respect, the difference was statistically significant (P<0.03).

Table 1 shows the comparison of iron profile among the groups. The mean values of serum iron were 52.38 and 64.50 microg/dL, respectively, for the case and control groups (p<0.02). TIBC levels in obese and non-obese children were 434.80 and 382.28 microg/dL (p<0.008). The ferritin level in the study group was 70.56 versus 68.06 ng/ml in the control group (p=0.79).nIn obese children group, 8.3% (n=2) of children with iron deficiency had ferritin levels less than 15 while in control group 100% (n=14) of iron deficient children had ferritin levels below 15 (P<0.001).

Table 2 shows iron distribution on the basis of gender. In both groups, there were no statistically significant gender differences between iron-deficient and non iron-deficient children (P>0.05).

**Table I:** The comparison of iron profile between the case and the control group.

**TIBC**		**Ferritin**		**Serum Iron**		
**mean**	**SD**	**mean**	**SD**	**mean**	**SD**	
**434.80**	105.27	70.56	51.39	52.38	25.73	**Obese group**
**382.28**	88.316	68.06	44.06	64.50	28.66	**Non-obese group**
**0.008**		0.79		0.02		**PV**

**Table II:** Iron distribution on the basis of gender in the case and the control groups

**The control group**		**The case group**		
**Female**	**Male**	**Female**	**Male**	
**10** **(71.4)**	4 (28.6)	11(45.8)	13(54.2)	**With iron deficiency**
**17(47.2)**	19 (52.8)	11(42.3)	15)(57.7)	**Without iron deficiency**
**0.12**		0.80		**PV**

## Discussion

In recent decades, obesity has become the most important health concern (16-18). As technology develops children and adolescents spend more time in sedentary leisure activities such as watching television, playing video games, and using computers. On the other hand, foods with high energy content and low iron levels lead to increasing obesity and iron deficiency in children and adolescents (19). In Mashhad, a case-control study was performed by Ghaemi et al. from 2010 up to 2012 with the aim of figuring out the association between obesity and low levels of micronutrients such as iron. In their study, they evaluated a total of 280 obese children ranging from 2 to 16 years old and 280 children with normal weights within the same age range. Serum iron was lower than normal limits in 56.1% of obese children and in children with normal weight this figure was 10.4% (11). Cepeda-Lopez in Mexico showed that the risk of iron deficiency in obese Mexican women and children was 2-4 times higher of normal-weight individuals (20).

However Ghadiri et al. study in Iranian Population showed no difference in serum iron, TIBC, transferrin saturation index, and ferritin among normal weight, overweight, and obese persons. Excluding diabetic patients and maybe the well nutritional status of obese people, for example, intake of high iron foods can be proposed to explain their results (12). Different factors have been suggested to explain the association between obesity and iron deficiency such as genetic factors, physical inactivity leading to insufficient breakdown of myoglobin, and reduction in iron levels which released into the blood stream (10), impaired intestinal iron absorption (21-23), inadequate dietary iron intake, and increased iron requirements. Thus, low iron status in overweight individuals may be due to combination of nutritional and functional factors (24). Recently, researchers have pointed out that serum hepcidin is significantly elevated

 in obese persons compared to the people with normal weight (25-28). Some studies have shown that overweight children have higher circulating hepcidin concentrations and lower iron status in comparison with children who are not overweight (29-31). Hepcidin is a small peptide hormone which is released from liver and adipose tissue cells (32). It inhibits iron uptake by enterocytes (33) and down-regulates non-heme iron release from macrophages (34). A case-control study by Sanad et al. in Egypt aimed to compare the serum hepcidin levels in children suffering from iron deficiency anemia (both obese and non-obese) with a control group of non-obese and healthy children. This study showed that considering control group, serum hepcidin was significantly lower in non-obese children with iron deficiency anemia and significantly higher in obese children with iron deficiency anemia (35). In Italy, in the study by Amato et al. hepcidin levels were higher in obese children compared to the controls which lead to reduceing iron absorption. The participants were then subjected to a 6-month weight loss program. After the program, all children reduced their body mass index and it was found that BMI reduction is associated with hepcidin reduction, potentially improving iron status and absorption (36). Thus in previous studies, this substance have had higher levels in obese children and considered as a predisposing factor for iron deficiency in overweight children.

In Mujica-Coopman study, dietary iron absorption was compared with iron status in obese, overweight and normal women of childbearing age. although no relationship between BMI and iron status was observed, obese women displayed lower iron absorption compared with overweight and normal weight women, possibly due to subclinical inflammation associated with obesity (37). This study assessed childbearing age women who are usually offered preconception care counseling (regardless of intent to pregnancy) in order to find out their iron deficiency during the routine preconception visits and present necessary treatments. The findings of present study could affect the results of previous study.

In our study, mean serum iron levels was lower among obese children compared to the controls. However, ferritin concentrations were similar in both groups. 91.7% of obese children with iron deficiency had also ferritin levels greater than 15. In Sharma et al. study, children with higher BMIs had lower serum iron levels; ferritin concentrations were similar in both the obese and the normal weight children (38) which is consistent with our study results. In a review study, Zafon refers to abnormally higher concentrations of ferritin in obese patients, likewise, and suggests that abnormal ferritin concentrations can be explained by chronic inflammation rather than by iron overload (21). In the study by Muschonis et al. in Greece, the association between being overweight and iron deficiency was examined in children aged between 9-13 years old and it was found that the prevalence of iron deficiency anemia was higher in obese children. However, in spite of this serum ferritin was significantly higher in obese children compared to their normal weight peers (39). Ferritin is an acute phase protein that may be increased during inflammation (40). Hence, unlike the usual non-inflammatory state where ferritin levels below 15 is considered as iron deficiency, in the case of inflammation and infection, values less than 30 will be interpreted as iron deficiency since in these cases (i.e. inflammation and infection) ferritin levels will be elevated (15). In their study, Gartner et al. have shown that obesity increases the inflammation-related hematological indices (41). Other studies, as well, have shown that obesity is accompanied by a state of low grade and chronic inflammation (42-45). It seems that serum ferritin levels are naturally higher in obese people due to inflammatory state caused by obesity. Therefore, similar to the cases of infection that the least normal level of ferritin is 30 micrograms per deciliter, in the cases of obesity higher serum ferritin levels should be considered as normal reference values.

## Conclusion

Given the increasing prevalence of obesity among children and adolescents and the prevalence of iron and other micronutrients deficiencies in obese children, it appears that children with elevated BMI should be screened for iron deficiency. It is also suggested that higher levels of ferritin should be considered as normal reference values in obese people. Because of the inflammatory state caused by obesity, determining its ranges requires further studies with larger sample sizes.
